# Deletion of S-Layer Associated Ig-Like Domain Protein Disrupts the *Lactobacillus acidophilus* Cell Surface

**DOI:** 10.3389/fmicb.2020.00345

**Published:** 2020-03-17

**Authors:** Courtney Klotz, Yong Jun Goh, Sarah O’Flaherty, Brant Johnson, Rodolphe Barrangou

**Affiliations:** ^1^Genomic Sciences Graduate Program, North Carolina State University, Raleigh, NC, United States; ^2^Department of Food, Bioprocessing and Nutrition Sciences, North Carolina State University, Raleigh, NC, United States; ^3^Microbiology Graduate Program, North Carolina State University, Raleigh, NC, United States

**Keywords:** *Lactobacillus*, probiotic, S-layer, cell surface, S-layer associated protein, Ig-like domain

## Abstract

Bacterial surface-layers (S-layers) are crystalline arrays of repeating proteinaceous subunits that coat the exterior of many cell envelopes. S-layers have demonstrated diverse functions in growth and survival, maintenance of cell integrity, and mediation of host interactions. Additionally, S-layers can act as scaffolds for the outward display of auxiliary proteins and glycoproteins. These non-covalently bound S-layer associated proteins (SLAPs) have characterized roles in cell division, adherence to intestinal cells, and modulation of the host immune response. Recently, IgdA (LBA0695), a *Lactobacillus acidophilus* SLAP that possesses a Group 3 immunoglobulin (Ig)-like domain and GW (Gly-Tryp) dipeptide surface anchor, was recognized for its high conservation among S-layer-forming lactobacilli, constitutive expression, and surface localization. These findings prompted its selection for examination within the present study. Although IgdA and corresponding orthologs were shown to be unique to host-adapted lactobacilli, the Ig domain itself was specific to vertebrate-adapted species suggesting a role in vertebrate adaptation. Using a counterselective gene replacement system, *igdA* was deleted from the *L. acidophilus* NCFM chromosome. The resultant mutant, NCK2532, exhibited a visibly disrupted cell surface which likely contributed to its higher salt sensitivity, severely reduced adhesive capacity, and altered immunogenicity profile. Transcriptomic analyses revealed the induction of several stress response genes and secondary surface proteins. Due to the broad impact of IgdA on the cellular physiology and probiotic attributes of *L. acidophilus*, identification of similar proteins in alternative bacterial species may help pinpoint next-generation host-adapted probiotic candidates.

## Introduction

Lactic acid bacteria (LAB) are a diverse group of Gram-positive, non-sporulating, microaerophilic organisms classified by the production of lactic acid as their signature fermentation end product (Claesson et al., 2007). Within this order are several industry-relevant genera including *Lactococcus*, *Enterococcus*, *Oenococcus*, *Pediococcus*, *Streptococcus*, *Leuconostoc*, and many *Lactobacillus* species (Makarova et al., 2006). For centuries these organisms have been harnessed for food and feed fermentations, but several species, mainly *L. acidophilus*, *L. casei*, *L. johnsonii*, *L. plantarum*, *L. reuteri*, *L. rhamnosus*, *L. salivarius*, and *L. bulgaricus*, have also gained notoriety for their probiotic attributes (Johnson and Klaenhammer, 2014). Probiotics are generally defined as “live microorganisms which when administered in adequate amounts confer a health benefit on the host” (Hill et al., 2014). Research has begun to hone in on the molecules and genes associated with these health-promoting properties chiefly to augment next-generation probiotic screening efforts (Lebeer et al., 2008; Siciliano et al., 2019). Of particular interest are extracellular proteins, such as surface-layers (S-layers), which can directly interact with and influence the host gastrointestinal tract (Kleerebezem et al., 2010; Lebeer et al., 2010).

S-layers are two-dimensional crystalline arrays that form the outermost coating of some cell envelopes. They are comprised of repeating subunits, S-layer proteins (Slps), which are inherently driven to self-assemble into a regularly spaced lattice structure (Fagan and Fairweather, 2014). S-layers are prevalent in archaea, but have also been detected on the exteriors of both Gram-positive and Gram-negative bacteria (Sleytr and Beveridge, 1999; Albers and Meyer, 2011). As one of the most abundant molecules produced by the cell, Slps play pivotal roles in growth and survival, cell integrity, and interactions with the host and its immune system (Sleytr and Beveridge, 1999; Sara and Sleytr, 2000; Hynonen and Palva, 2013; Sleytr et al., 2014). S-layers can also act as scaffolds for the external display of auxiliary proteins and glycoproteins which confer supplementary functionalities (Fagan and Fairweather, 2014; Siciliano et al., 2019). The S-layer associated proteins (SLAPs) of *L. acidophilus* have been linked to diverse roles in cell division, intestinal adhesion, and host immunomodulation (Altermann et al., 2004; Johnson et al., 2013, 2017; Hymes et al., 2016; Johnson and Klaenhammer, 2016).

Recently, while mining the SLAPs of S-layer-forming lactobacilli, four highly conserved gene regions were identified (Johnson et al., 2015). Contained within these regions were two peptidoglycan hydrolases (Johnson and Klaenhammer, 2016), a fibronectin-binding protein (Hymes et al., 2016), and two cell division proteins, one of which was CdpA (Altermann et al., 2004). These groups of genes tended to be highly expressed, exhibited synteny across lactobacilli genomes, and were predominantly localized near the origin of replication (Johnson et al., 2015). However, the fourth region, encompassing a Group 3 bacterial immunoglobulin (Ig)-like domain (Big_3) protein with a GW (Gly-Tryp) dipeptide surface anchor, clustered separately and has yet to be functionally examined. Bacterial Ig-like domains, frequently observed in cell surface proteins, have been shown to mediate cell-to-cell recognition and surface receptor functions (Luo et al., 2000; Pavkov et al., 2008). Variants of this domain have been identified in several Slps including the Big_2 domain within the *Bacillus anthracis* surface array protein (Sap) and the Big_3 domain in cell wall binding protein, Cwp12, of *Clostridium difficile* (Fagan and Fairweather, 2014).

The Ig domain-containing SLAP of *L. acidophilus* NCFM (LBA0695), which we have designated IgdA, was recently recognized for its high constitutive expression on the cell exterior (Klotz et al., 2017). Its conservation among S-layer-forming lactobacilli, compelling domain architecture, and high surface abundance, prompted its selection for further investigation. In the following study we provide evidence that IgdA plays a significant role in cell surface maintenance and, indirectly, host adaptation. Our *in silico* analyses revealed that *igdA* and corresponding orthologs were unique to host-adapted lactobacilli, while the Ig domain itself was specific to vertebrate-adapted species. To further explore these findings, *igdA* was deleted from the *L. acidophilus* chromosome using a pORI-based *upp* counterselective gene replacement system (Goh et al., 2009). The *igdA* mutant strain, NCK2532, exhibited a visibly disrupted cell surface which likely contributed to its increased salt sensitivity, altered immunogenicity profile, and severely reduced adhesion to Caco-2 intestinal cells, extracellular matrices, and mucin *in vitro*.

## Materials and Methods

### Mapping *igdA* to a Lactobacilli Phylogenetic Tree

Phylogenetic analyses were used to gain insight into the occurrence of *igdA* within a curated dataset of 170 *Lactobacillus* genomes downloaded from NCBI (Supplementary Table S1; O’Flaherty et al., 2018). A cladogram was constructed using a previously described method based on the nucleotide sequence of the pyruvate kinase (Pyk) enzyme (Brandt and Barrangou, 2016; O’Flaherty et al., 2018). Briefly, *pyk* and *igdA* orthologs were identified and extracted from 170 genomes, encompassing 170 different *Lactobacillus* species, via Geneious annotation and extraction workflows (Kearse et al., 2012). The 170 *pyk* nucleotide sequences were then aligned in CLC Genomics Workbench (Qiagen) and the output was used to construct a lactobacilli phylogenetic tree. The CLC Genomics metadata feature was employed to map the presence or absence of Slps and *igdA*, as well as species lifestyle. S-layer presence was determined using the UniProt annotation tool suite to search *Lactobacillus* proteomes present within the UniProt database (The UniProt Consortium, 2017). Lifestyle information was adapted from Duar et al. (2017).

To construct a phylogenetic tree based on the IgdA amino acid sequences, Geneious software (Kearse et al., 2012) was used to translate the *igdA* nucleotide sequences identified above. Translated sequences were imported and aligned in CLC Genomics Workbench (Qiagen) and the output was used to construct an unrooted radial tree. The Duar et al. (2017) lifestyle roles were again mapped to the tree, in addition to GW and Big_3 protein domains which were predicted using the Geneious InterProScan plugin (Quevillon et al., 2005).

### Bacterial Strains and Growth Conditions

The bacteria and plasmids used in this study are listed in Table 1. *Lactobacillus* strains were propagated in de Man, Rogosa and Sharpe (MRS) broth (Difco Laboratories, Detroit, MI) statically under ambient atmospheric conditions or on MRS agar plates [1.5% (w/v); Difco] anaerobically, and incubated at either 37°C, or 42°C for pTRK669 plasmid elimination (Russell and Klaenhammer, 2001). Recombinant *L. acidophilus* strains were selected in the presence of 2 μg/mL erythromycin (Sigma-Aldrich, St. Louis, MO, United States) and/or 2–5 μg/mL chloramphenicol (Sigma). Selection of plasmid-free double recombinants was performed on a semidefined agar medium containing 2% (wt/vol) glucose (GSDM) (Kimmel and Roberts, 1998) and 100 μg/mL 5-fluorouracil (5-FU; Sigma), as previously described (Goh et al., 2009). *Escherichia coli* EC101 was grown in brain heart infusion (BHI) broth (Difco) with aeration at 37°C in the presence of 40 μg/mL kanamycin (Sigma-Aldrich). Recombinant *E. coli* EC101 cells containing pTRK935-based plasmids were selected using 150 μg/mL erythromycin (Goh et al., 2009).

**TABLE 1:** Bacterial strains, plasmids, and PCR primers used in this study.

Strain, plasmid, or primer	Genotype or characteristics^a^	References
**Strains**		
*Lactobacillus acidophilus*		
NCK56 (NCFM)	Human intestinal isolate	Sanders and Klaenhammer, 2001
NCK1909 (Δ*upp*)	NCFM with a 315 bp in-frame deletion within the *upp* gene (*lba0770*); background/parent strain for NCFM deletion mutants	Goh et al., 2009
NCK1910	NCK1909 harboring the *repA* helper plasmid pTRK669; host for pORI-based counterselective integration vector	Goh et al., 2009
NCK2532 (Δ*igdA)*	NCK1909 with a 1,587 bp in-frame deletion within the *lba0695* gene	This study
*Escherichia coli*		
EC101	RepA^+^ JM101; Km^r^; *repA* from pWV01 integrated into chromosome; cloning host for pORI-based plasmids	Law et al., 1995
NCK1911	EC101 host harboring pTRK935 integration vector	Goh et al., 2009
NCK2531	EC101 host harboring pTRK1127 recombinant plasmid	This study
**Plasmids**		
pTRK669	Ori (pWV01), Cm^r^, RepA^+^, thermosensitive	Russell and Klaenhammer, 2001
pTRK935	pORI *upp*-based counterselective integration vector, Em^r^	Goh et al., 2009
pTRK1127	pTRK935 harboring a mutated copy *lba0695* gene cloned into *Bam*HI/*Sac*I site	This study
**Primers**		
Construction of *igdA* (*lba0695*) deletion mutant		
0695BamHIF	GATCTAGGATCCGTTGATCTTCTTACGACTCTTC	This study
0695R	CAAGACTATCCTCCATAATCTCAT	This study
0695Soe	GATTATGGAGGATAGTCTTGCGTGCTGAATTATTTGAAAGTAAT	This study
0695SacIR	GATCTAGAGCTCCCTTAGTAATTAGTATTGATGCTCC	This study
PCR analysis and DNA sequencing of deletion targets		
0695up	CATATTCCTTAGCTTCTTCAGCAG	This study
0695dw	GCACCTGCAATTAATCCTCATG	This study

*^a^For primers, the 5′-to-3′ sequences are given and restriction enzyme sites are underlined.*

### DNA Manipulations and Transformation

Genomic DNA was isolated using the ZR Fungal/Bacterial DNA MiniPrep Kit (Zymo Research, Irvine, CA, United States). Plasmid DNA was isolated using the QIAprep Spin MiniPrep Kit (Qiagen, Hilden, Germany). Restriction enzymes, Quick Ligase, and Q5 High-Fidelity 2X Master Mix (New England Biolabs, Ipswich, MA, United States) were used for cloning purposes, while Choice Taq Blue DNA polymerase (Denville Scientific, South Plainfield, NJ, South Plainfield) was employed for PCR screening of recombinants. Amplicons were visualized on 1% (wt/vol) agarose gels, then extracted using the QIAquick Gel Extraction Kit (Qiagen). DNA sequencing was performed by Eton Bioscience, Inc. (Research Triangle Park, NC, South Plainfield).

### Chromosomal Deletion of *igdA*

The *lba0695* gene, encoding S-layer associated protein IgdA, was deleted from the *L. acidophilus* NCFM genome via a pORI-based *upp* counterselective gene replacement system (Goh et al., 2009). Briefly, an in-frame deletion was constructed by PCR amplifying flanking regions up and downstream of the deletion target (Table 1). The resultant purified products were joined by splicing using overlap extension PCR (SOE-PCR), then amplified to create the deletion genotype. The SOE-PCR product was cloned within the *Bam*HI and *Sac*I sites of the pTRK935 integration vector, then transformed into *E. coli* EC101. The recombinant plasmid (confirmed by DNA sequencing) was electroporated into *L. acidophilus* NCK1910 containing the pTRK669 temperature sensitive helper plasmid (Table 1). Recovery of single- and double-crossover recombinants was performed as previously described (Goh et al., 2009). The absence of *lba0695* was confirmed by sequencing the entirety of both flanking regions.

### Lithium Chloride Isolation of Extracellular Proteins

Non-covalently attached surface proteins were isolated using a modified lithium chloride (LiCl) S-layer extraction protocol (Johnson et al., 2013). *L. acidophilus* strains were grown statically in 1 L of MRS broth (Difco) at 37°C for 16 h. All subsequent steps were conducted on ice or at 4°C. Bacterial cells were centrifuged at 3,220 × *g* for 10 min, then washed twice with cold (4°C) phosphate buffered saline (PBS, pH 7.4, Thermo Fisher Scientific, Waltham, MA, United States). Pellets were resuspended in 5 M LiCl (4°C) for 15 min with repeated agitation, then centrifuged at 7,441 × *g* for 10 min. Supernatants were transferred to Spectra/Por membrane tubing (6–8 kD, Spectrum Laboratories, Inc.) and dialyzed against cold distilled water (4°C) for 24 h with gentle stirring and frequent water changes. Overnight protein precipitates were centrifuged at 22,789 × *g* for 30 min, then resuspended in 1 M LiCl (4°C) for 15 min with repeated agitation. Suspensions were centrifuged at 22,789 × *g* for 30 min to separate major Slps from proteins associated with the S-layer. SLAP-containing supernatants were transferred to Spectra/Por membrane tubing (6–8 kD) and again dialyzed against cold distilled water (4°C) for 24 h with gentle stirring and frequent water changes. Precipitates were harvested via centrifugation at 22,789 × *g* for 30 min, then concentrated in 1 mL distilled water. Final suspensions were pelleted in 1.5 mL microcentrifuge tubes at 16,873 × *g* for 30 min. Both Slp and SLAP pellets were resuspended in 10% UltraPure SDS Solution (Thermo Fisher Scientific). Protein was quantified using a Micro BCA Protein Assay Kit (Thermo Fisher Scientific), then visualized with a Novex 10–20% Tris–glycine mini gel (Invitrogen) stained with AcquaStain (Bulldog Bio).

### Growth Analysis Under Stress Conditions

Bacterial growth was assessed under various stress culture conditions (Khaleghi et al., 2010; Grosu-Tudor et al., 2016). Overnight *L. acidophilus* cultures were used to inoculate 96-well microplates (Corning Costar, Corning, NY) containing 200 μl of MRS broth or MRS broth supplemented with 2.5% (w/v) NaCl (Fisher Scientific, Hampton, NH, United States), 0.2% (w/v) porcine bile (Sigma) or 0.5% (w/v) oxgall (Difco). Plates were sealed with clear adhesive film then incubated at 37°C in a Fluostar Optima microplate reader (BMG Labtech, Cary, NC, United States). The culture turbidity was recorded at OD_600_ every hour for 30 h. To validate results obtained with the plate reader, strains grown in MRS and MRS containing 2.5% NaCl were also enumerated on MRS solid media. Overnight bacterial cultures were used to inoculate broth treatments. Aliquots were taken at 0, 4, 6, 8, 10, and 24 h and plated on MRS agar to obtain viable cell counts.

### Examination of Mutant Cellular Morphology

Phase contrast microscopy was used to visualize *L. acidophilus* strains during the 24 h growth curves described in the section above. Observed irregularities were further investigated via electron microscopy. Mutant and parent cells were again grown to log (6 h) and early stationary (12 h) growth phases in MRS broth and MRS broth containing 2.5% NaCl. Cells were fixed in a solution of 3% glutaraldehyde in 0.1 M sodium cacodylate (pH 5.5) and stored at 4°C. Fixed cells were processed for scanning electron microscopy (SEM) and transmission electron microscopy (TEM) by the CALS Center for Electron Microscopy (CEM) at North Carolina State University. SEM images were acquired with a JEOL JEM-5900LV SEM (JEOL United States, Peabody, MA, United States) at 15 kV, while TEM grids were viewed in a JEOL JEM-1200EX TEM (JEOL United States) at 80 kV with images digitally acquired using a Gatan Model 795 ES 1000W Erlangshen CCD camera. High-resolution SEM images were acquired using an FEI Verios 460L Field Emission Scanning Electron Microscope located at the Analytical Instrumentation Facility (AIF) also housed at North Carolina State University.

Flow cytometry was used to survey changes to cell size and shape on a population level. Mutant and parent cells were again grown to log (6 h) and early stationary (12 h) growth phases in MRS broth as well as MRS broth containing 2.5% NaCl. Cultures were centrifuged at 3,220 × *g* for 10 min, then washed and resuspended in PBS (Thermo Fisher Scientific). Forward and side scattering patterns were acquired using a CytoFLEX Flow Cytometer instrument (Beckman Coulter, Brea, CA, United States) located at the CVM Flow Cytometry and Cell Sorting core facility (North Carolina State University). Data analysis was performed with the CytExpert software (Beckman Coulter).

### Adhesion to Mucin and Extracellular Matrices (ECM)

Mucin and ECM binding assays were adapted from a previously described protocol (Goh and Klaenhammer, 2010). Adhesion substrates were dissolved in various buffers to obtain a final concentration of 10 mg/mL. Mucin (Type III from porcine stomach, Sigma) was suspended in PBS (Thermo Fisher Scientific), while fibronectin (from human plasma, Sigma), collagen (type IV from human cell culture, Sigma), and laminin (from Engelbreth-Holm-Swarm murine sarcoma/basement membrane; Sigma) were solubilized in 50 mM carbonate-bicarbonate buffer (pH 9.6, Sigma). Wells of Nunc Maxisorp 96-well microplates (Sigma) were coated with 100 μl of solubilized substrate then incubated overnight at 4°C. The following day, coated wells were washed twice with PBS (pH 7.4), then blocked with 150 μl of 2% bovine serum albumin (BSA) solution (Sigma) for 2 h at 37°C. Excess BSA was removed by washing twice with PBS (pH 7.4). *Lactobacillus* strains, grown to early stationary phase (12 h) in MRS broth, were pelleted (3,220 × *g*, 10 min) at room temperature then washed once and resuspended in PBS (pH 5). Cell concentrations were adjusted to ∼1 × 10^8^ CFU/mL, then transferred (100 μl) to coated wells or enumerated on MRS plates to obtain initial counts. Following a 1 h incubation at 37°C, unattached cells were removed by gently washing wells five times with 200 μl/well of PBS (pH 5). Adhered cells were then recovered by adding 100 μl of 0.5% Triton X-100 solution (Fisher Scientific, prepared in PBS) and agitating on an orbital shaker (150 rpm) for 15 min. Cell suspensions were transferred to 900 μl of 0.1X MRS broth and enumerated on MRS media. Relative adherence percentages were calculated by standardizing the parent strain adherence to 100%. Assays were performed in biological triplicate. Significance was calculated using a Student’s *t*-test.

### Adhesion to Caco-2 Intestinal Cells

The Caco-2 intestinal cell line was purchased from American Type Culture Collection. Cells were grown in minimum essential medium (MEM, Thermo Fisher Scientific) containing Earle’s salts and 2 mM L-glutamine supplemented with 10% Fetal Bovine Serum (FBS, Thermo Fisher Scientific), MEM non-essential amino acids (Thermo Fisher Scientific), MEM sodium pyruvate (Thermo Fisher Scientific) and antibiotic/antimycotic solution (Thermo Fisher Scientific). Cells were split approximately every 3–4 days before reaching confluence and medium was replaced every other day. Cells were maintained in 75 cm^2^ cell culture flasks (Falcon) housed in a 37°C humidified atmosphere with 5% CO_2_.

Adhesion assays were performed as previously described by Goh et al. (2009) with minor modifications. Cells were allowed to reach confluence and differentiate for 21 days in 12-well plates with a seeding density of 1.6 × 10^5^ cells/well. On the day of experiment, confluent and fully differentiated Caco-2 cells were rinsed twice with PBS buffer and culture medium was replaced with 2 mL antibiotic/antimyotic free medium. Early stationary growth phase (12 h) *L. acidophilus* strains were pelleted (3,220 × *g*, 10 min), washed twice with PBS, and suspended in antibiotic/antimyotic free cell culture medium at a concentration of ∼1 × 10^8^ CFU/mL. Suspensions were enumerated on MRS plates to confirm projected cell counts. Strains were then co-incubated (1 mL) with Caco-2 monolayers for 1 h, followed by five PBS washes to remove unadhered bacterial cells. Adhered cells were recovered by disrupting the monolayer with 1 mL of 0.05% Triton X-100 (Fisher Scientific, prepared in PBS). Cell suspensions were transferred to 4.5 mL of 0.1X MRS broth and enumerated on MRS media. Relative adherence percentages were calculated by adjusting the parent strain to 100%. Assays were performed in biological quadruplicate. Significance was calculated using a Student’s *t*-test.

### Bacteria/DC Co-incubation and Cytokine Measurement

Bone marrow-derived C57BL/6 murine immature dendritic cells (DCs) were purchased from Astarte-Biologics (Bothell, WA, United States) and preserved in liquid nitrogen. Bacterial co-incubation assays were performed as previously described, with minor modifications (Johnson et al., 2013). On the day of the experiment, DCs were thawed in a 37°C water bath then transferred to a 50 mL conical tube containing 100 μg of DNase I (Stemcell Technologies). Cells were suspended in 25 mL RPMI 1640 medium (Thermo Fisher Scientific) with 10% FBS (Thermo Fisher Scientific), then centrifuged twice for 15 min at 200 × *g* to wash. Pelleted DCs were again suspended in 25 mL RPMI 1640 + 10% FBS + 100 μg of DNase I. Viable cells were quantified using Trypan Blue dye (Sigma) with the Invitrogen Countess, per manufacturer’s instructions. Based on the viable count, cells were diluted to a final concentration of 10^6^/mL in RPMI 1640 + 1% FBS + 100 μg of DNase I media and aliquoted (100 μl per well) into round bottom polypropylene 96-well plates. Plates were held in a 37°C humidified atmosphere with 5% CO_2_ while bacterial cells were prepared.

Early stationary growth phase (12 h) *L. acidophilus* strains were centrifuged at 9,600 × *g*, then washed and resuspended in PBS (Thermo Fisher Scientific). Based on the OD_600_, cell concentrations were adjusted to 5 × 10^7^ CFU in 1 mL RPMI 1640 + 10% FBS + 100 μg streptomycin (Sigma). An aliquot of the suspension was diluted in 0.1X MRS and enumerated on MRS media to verify cell counts. Bacterial suspensions (200 μl) were co-incubated with DCs for 24 h in a 37°C humidified atmosphere with 5% CO_2_ then centrifuged (3,220 × *g*, 10 min, 4°C) to collect supernatant for cytokine quantification. Supernatants were stored at −80°C until processed. Cytokine measurements for tumor necrosis factor α (TNF-α) and interleukin (IL)-6, IL-10, and IL-12 were obtained using Single-Analyte ELISArray kits (Qiagen) according to manufacturer’s instructions. Assays were performed in biological triplicate. To reduce random error, replicates were treated as a blocking factor. Significance of block centered data was analyzed using a Student’s *t*-test.

### RNA Extraction, Sequencing, and Analysis

Total RNA was isolated from the *L. acidophilus* parent strain and *igdA*-deficient mutant propagated in MRS broth and MRS broth containing 2.5% NaCl. Cells were grown statically under ambient atmospheric conditions for 6 h, pelleted by centrifugation (3,220 × *g*, 5 min, RT), then flash frozen and stored at −80°C. For RNA extraction, frozen pellets were resuspended in 1 mL of TRI reagent (Thermo Fisher Scientific) then transferred to 1.5-mL screwcap tubes containing 0.1-mm glass beads (BioSpec Products, Inc., Bartlesville, OK, United States). Cells were disrupted for six 1 min cycles, each paired with a 1 min ice incubation, using a Mini-Beadbeater 16 homogenizer (BioSpec Products). Total RNA was isolated using a Zymo Direct-Zol RNA Miniprep kit (Zymo Research) as per manufacturer’s instructions, followed by a Turbo DNAse (Thermo Fisher Scientific) treatment and further purification using an RNA Clean and Concentrator-5 kit (Zymo Research). The RNA quality was verified using an Agilent 2100 Bioanalyzer (Agilent Technologies, Santa Clara, CA), while the absence of genomic DNA was confirmed with PCR using *L. acidophilus* NCFM-specific primers (Theilmann et al., 2017). Library preparation and RNA sequencing were conducted by the High-Throughput Sequencing and Genotyping Unit of the Roy J. Carver Biotechnology Centre housed at the University of Illinois (Urbana-Champaign, IL, United States). The RNA-seq reads were filtered and mapped to the *L. acidophilus* NCFM reference genome using Geneious software (Kearse et al., 2012) with default settings. Expression levels were compared with the DESeq2 package (Love et al., 2014). Enrichment analyses were performed using the DAVID Bioinformatics Resource 6.8 Functional Annotation Tool (Huang et al., 2009b, a) and Cluster of Orthologous Groups (COGs) were assigned with EggNOG 5.0 (Huerta-Cepas et al., 2016). The transcriptomic datasets generated in this study are available in the National Center for Biotechnology database under BioProject ID PRJNA576881.

## Results

### Occurrence of *igdA* Within Host-Adapted S-Layer-Forming Lactobacilli

**FIGURE 1:**
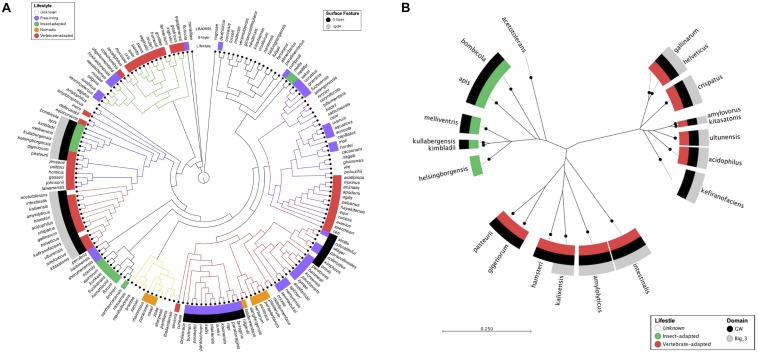
Mapping *igdA* and *igdA* domains to lactobacilli phylogenetic trees. A phylogenetic tree constructed using the pyruvate kinase gene sequences of 170 *Lactobacillus* strains. The inner metadata layer maps lifestyle designations modified from Duar et al. (2017). The outer metadata layers map the presence/absence of S-layer proteins and *igdA* orthologs (>55% similarity). Strain details can be found in Supplementary Table S1 **(A)**. An unrooted phylogenetic tree constructed using IgdA amino acid sequences. The inner metadata layer maps lifestyle designations modified from Duar et al. (2017). The outer metadata layers map GW and Big_3 domain presence **(B)**.

The presence of Slps and *igdA* orthologs was mapped to a lactobacilli phylogenetic tree constructed based on the *pyk* gene sequence (Figure 1A). The specific strains used for this study and their isolation sources are listed in Supplementary Table S1. Among the 170 evaluated genomes, 40 were shown to possess S-layers. Although *igdA* was unique to S-layer-forming strains, only half contained an *igdA*-like sequence with >55% similarity. S-layers were present in vertebrate-adapted, insect-adapted, and free-living species, but absent from nomadic species – i.e., organisms lacking specific environmental genetic adaptations (Martino et al., 2016). The *igdA* gene was only found in certain vertebrate-adapted and insect-adapted species.

An unrooted radial tree was constructed using the IgdA amino acid sequences (Figure 1B). In addition to species lifestyle (mentioned above), Big_3 and GW domain presence were also mapped onto the tree. The protein sequences formed three distinct clusters: two vertebrate-adapted groups and one insect-adapted group. Although the GW anchor was present in both insect-adapted and vertebrate-adapted species, the Big_3 domain was confined to vertebrate-adapted species.

### Deletion of *igdA* From the *L. acidophilus* NCFM Genome

**FIGURE 2:**
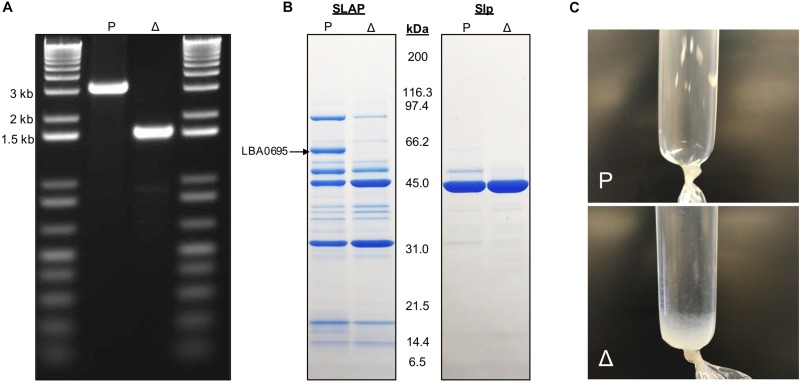
Images depicting the deletion of *igdA*. PCR was used to confirm removal of 1,587 bp within the *lba0695* locus using primers that flanked the deletion region **(A)**. Surface proteins were isolated using a LiCl extraction protocol and visualized on a Tris–glycine gel **(B)**. Surface protein isolated after a 5M LiCl treatment and dialysis against cold distilled water for 24 h **(C)**. P, NCK1909 parent strain, Δ, NCK2532 harboring the *igdA* deletion.

A pORI-based *upp* counterselective gene replacement system (Goh et al., 2009) was used to generate a 1,587 bp (96%) in-frame deletion within the *lba0695* locus of the *L. acidophilus* NCFM genome. The mutant strain was designated NCK2532 (Table 1). The deletion was detected via PCR (Figure 2A) and further confirmed by Sanger sequencing. Following LiCl treatment, the absence of the LBA0695 protein was visualized on a Tris–glycine gel (Figure 2B). A marked increase in abundance of surface protein was noted in the S-layer fraction of the NCK2532 mutant compared to the parent after 5 M LiCl treatment and dialysis (Figure 2C). In contrast, the typical starting volume of cells had to be scaled-up five times to obtain enough SLAP for protein gel analysis (Figure 2B).

### Inhibition of Δ*igdA* Growth and Survival by High Salt Conditions

**FIGURE 3:**
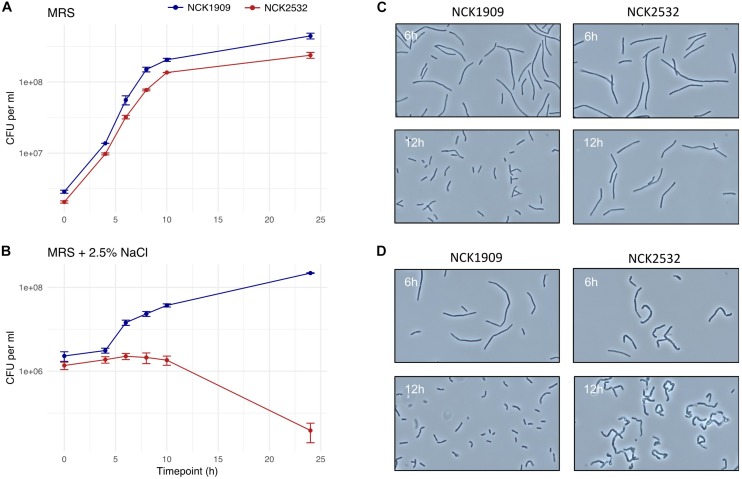
Growth analysis of the *igdA* deletion mutant compared to the parent strain in MRS and high salt conditions. Viable cell counts of NCK1909 and NCK2532 (Δ*igdA*) were recorded over the course of 24 in MRS broth **(A)** and MRS broth supplemented with 2.5% NaCl **(B)**. This data is complemented by phase contrast microscopy images depicting strain cellular morphology at 6 and 12 h in MRS broth **(C)** and MRS broth + 2.5% NaCl **(D)**.

The turbidity of the parent and mutant strains was monitored over the course of 30 h in MRS broth, as well as MRS broth containing 2.5% NaCl, 0.2% porcine bile, or 0.5% oxgall (Supplementary Figure S1). The only discernable difference occurred in the high salt medium. The growth of the *igdA*-deficient mutant was consistently hindered by NaCl presence, though this effect only becomes apparent at ≥2% (data not shown). A direct correlation between OD and CFU/mL does not always exist (O’Flaherty and Klaenhammer, 2010), therefore MRS and high salt treatments were also enumerated on MRS agar over the course of 24 h (Figures 3A,B). The cell counts of MRS cultures were not strikingly different, though NCK2532 routinely had a lower final CFU/mL in comparison to the NCK1909 parent strain. In contrast, the salt stressed mutant exhibited impaired growth within the first 6 h before undergoing a nearly four log reduction at 24 h.

### Alterations to Δ*igdA* Cellular Morphology and Surface Structure

**FIGURE 4:**
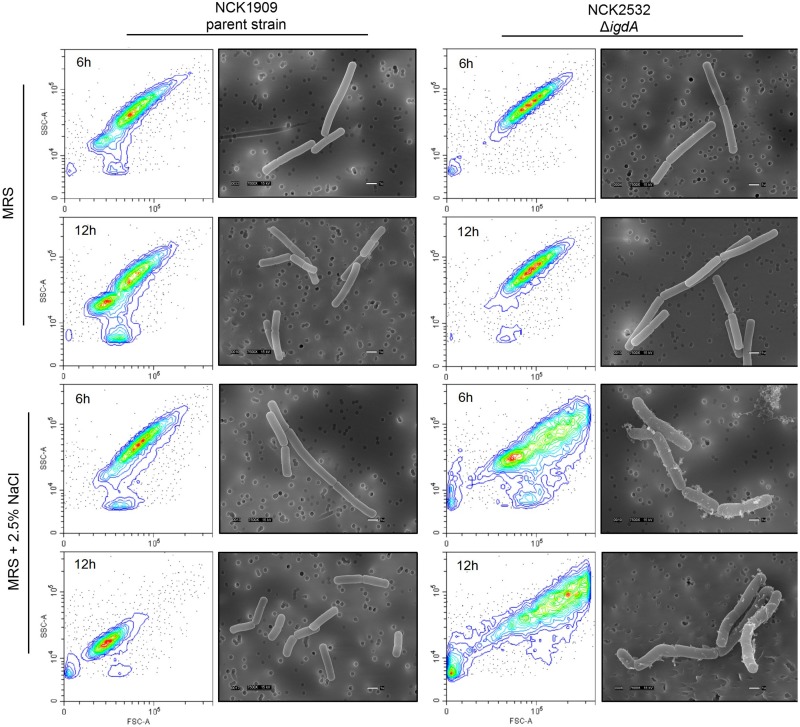
Cellular morphologies of the parent strain and *igdA* mutant were evaluated using flow cytometry and scanning electron microscopy. Bacteria were grown statically in MRS broth or MRS broth supplemented with 2.5% NaCl for 6 and 12 h.

**FIGURE 5:**
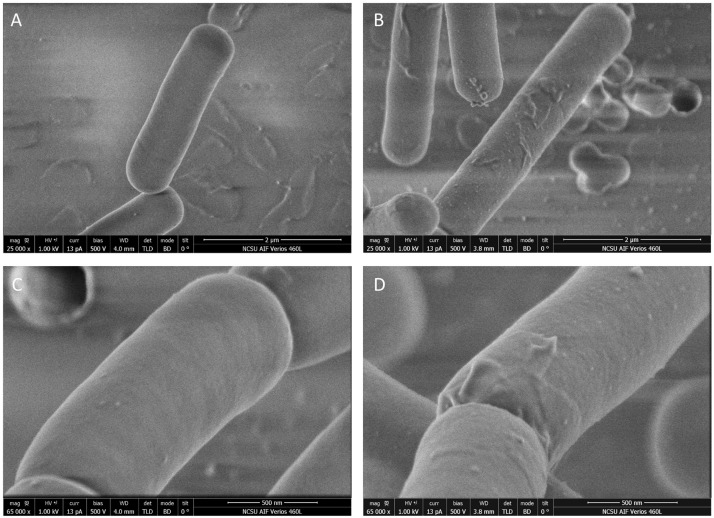
Images of NCK1909 and NCK2532 (Δ*igdA*) cell surfaces obtained using high-resolution scanning electron microscopy. Low magnifications images (25,000x) of NCK1909 **(A)** and NCK2532 **(B)**. High magnification images (65,000x) of NCK1909 **(C)** and NCK2532 **(D)**. Bacteria were grown statically in MRS broth for 12 h.

Phase contrast microscopy was used to visualize *L. acidophilus* strains during growth in MRS broth and MRS broth containing 2.5% NaCl. The 6 and 12 h time points are shown in Figures 3C,D. In MRS broth, the *igdA*-deficient NCK2532 cells were noticeably elongated, particularly during stationary growth phase (Figure 3C). Under high salt conditions, mutant cells became misshapen at 6 h and developed a kinked morphology by 12 h (Figure 3D). These observed irregularities were further investigated via flow cytometry and SEM (Figure 4). In MRS the Δ*igdA* cells were again elongated, while under high salt conditions they exhibited drastically deteriorated exteriors. TEM and high-resolution SEM were used to obtain high-resolution images of the *L. acidophilus* cell surfaces. Although TEM images did not depict any obvious differences between the two strains (Supplementary Figure S2), high-resolution SEM exposed an acutely disrupted mutant cell surface structure (Figure 5).

### Adherence Deficiencies of Δ*igdA in vitro*

**FIGURE 6:**
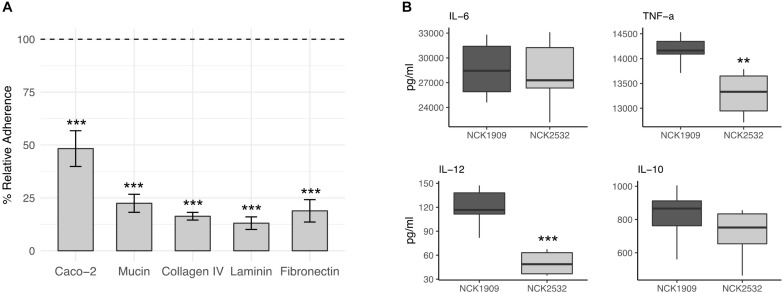
Effects of the *igdA* deletion on the adhesive capacity and immunogenicity of *Lactobacillus acidophilus*. Relative adherence of the Δ*igdA* mutant was assessed using Caco-2 epithelial cells, mucin and major extracellular matrices (ECM). The adherence of the NCK1909 parent strain was standardized to 100% (dotted line). The data represent means of independent biological replicates. Error bars are standard error of the means **(A)**. The immunomodulatory profile of Δ*igdA* (light gray) compared to the parent strain (dark gray) was evaluated using a murine dendritic cell co-incubation assay. Cytokines IL-6, TNF-α, IL-12, and IL-10 were measured via ELISA. Co-incubation assays were performed in triplicate; box plots were constructed using block centered data **(B)**. For both plots, asterisks indicate statistical significance calculated using a Student *t*-test (^∗∗∗^*p* < 0.001, ^∗∗^*p* < 0.01).

In comparison to the parent, the Δ*igdA* mutant demonstrated significantly reduced adherence to all tested substrates as well as the Caco-2 intestinal cell line (Figure 6A). Among the evaluated ECMs, the NCK2532 mutant exhibited an 84, 87, and 81% relative decrease in adhesion to collagen, laminin, and fibronectin, respectively. Additionally, a 78% decrease in mucin adhesion was also observed. The reduction in relative Caco-2 binding was less severe, but still compelling at 51%. Both the mutant and parent strain exhibited similar susceptibility to diluted Triton X-100 alone, eliminating it as a potential source of variability (data not shown).

### Deletion of *igdA* Alters DC Cytokine Expression

The immunomodulatory properties of *igdA* deletion strain, NCK2532, were examined using an *in vitro* bacterial/murine DC co-incubation assay. The block centered data is plotted in Figure 6B. In comparison to the NCK1909 parent strain, the concentrations of pro-inflammatory IL-6 and anti-inflammatory IL-10 were relatively unchanged. In contrast, expression of pro-inflammatory molecules IL-12 and TNF-α were significantly repressed by co-incubation with the mutant strain.

### Transcriptomic Analysis of Δ*igdA* Reveals Upregulation of Multiple Stress Response Pathways and Alternative Surface Proteins

**FIGURE 7:**
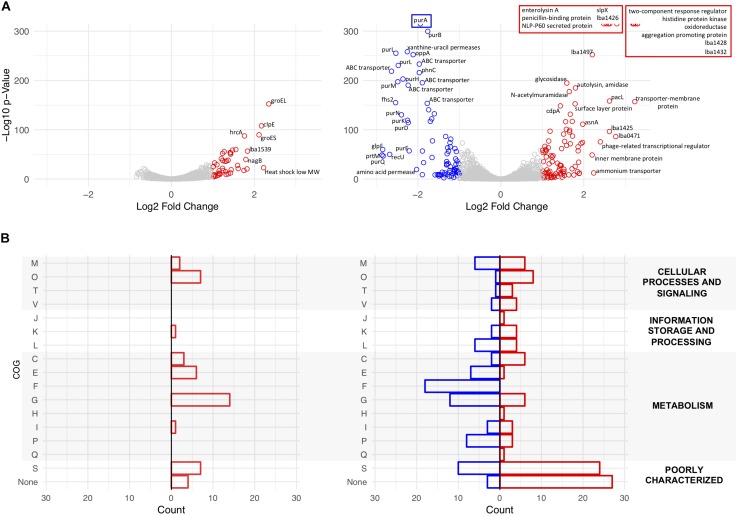
Transcriptional analysis of NCK1909 and NCK2532 (Δ*igdA*). Volcano plots depict Log2 fold change in expression of NCK2532 mutant compared to NCK1909 grown in MRS broth [**(A)**, left] and MRS broth +2.5% NaCl [**(A)**, right]. Significantly up and downregulated genes (*p*-value < 0.01 and |Log2 fold change| >1) are colored in red and blue, respectively. Clusters of Orthologous Groups (COGs) were assigned to significant genes using the EggNOG Database **(B)**. The categories are as follows: C, Energy production and conversion; E, amino acid transport and metabolism; F, nucleotide transport and metabolism; G, carbohydrate transport and metabolism; H, coenzyme transport and metabolism; I, lipid transport and metabolism; J, translation, ribosomal structure and biogenesis; K, transcription; L, replication, recombination and repair; M, cell wall/membrane/envelope biogenesis; O, post-translational modification, protein turnover, and chaperones; P, inorganic ion transport and metabolism; Q, secondary metabolites biosynthesis, transport, and catabolism; R, general function prediction only; S, function unknown; T, signal transduction mechanisms; V, defense mechanisms.

Transcriptome sequencing was used to analyze global transcription of the log phase Δ*igdA* mutant in comparison to the parent strain when cultured in either MRS broth or MRS broth containing 2.5% NaCl. Differentially expressed genes (*p*-value < 0.01 and |Log2 fold change| >1) are colored in red and blue, respectively (Figure 7A). The data used to create these plots can be found in Supplementary Tables S2, S3. To complement these results, COGs were assigned using the EggNOG Database (Huerta-Cepas et al., 2016; Figure 7B). Additionally, a previously curated list of the most abundant log phase SLAPs (Klotz et al., 2017), and corresponding fold change, is detailed in Table 2. In MRS, the absence of *igdA* triggered the significant upregulation of 44 genes, while none were downregulated (Figure 7A, left). Stress response genes *groEL*, *groES*, and *clpE* were the most significantly induced with a Log2 fold change >2. Assigned COGs supported this finding, but also revealed the upregulation of additional functions including several genes associated with the transport and metabolism of carbohydrates and amino acids (Figure 7B, left). All three Slp constituents (SlpA, SlpB, and SlpX), and predominant log phase SLAPs, were unaffected by the *igdA* deletion (Table 2).

**TABLE 2:** Differential expression of genes encoding surface associated proteins.

Gene	Predicted Function	Log2 Fold Change (*p*-value)	
		Δ*lba0695*	Δ*lba0695* + 2.5% NaCl
**SLP**			
*lba0169*	*slpA*	0.14 (0.048)	0.52 (0.000)
*lba0175*	*slpB*	0.23 (0.007)	0.55 (0.000)
*lba0512*	*slpX*	0.84 (0.000)	2.33 (0.000)
**SLAP**			
*lba1578*	Putative serine protease	0.21 (0.004)	−0.54(0.000)
*lba0695*	*igdA*	−8.76(0.000)	−10.13(0.000)
*lba1426*	Putative uncharacterized protein	0.32 (0.000)	2.54 (0.000)
*lba0889*	*eno* – Enolase	0.09 (0.183)	−0.51(0.000)
*lba0846*	*tig* – Trigger factor	−0.20(0.003)	−0.59(0.000)
*lba1567*	Aminopeptidase	0.11 (0.307)	−0.10(0.487)
*lba1162*	Asparagine–tRNA ligase	−0.24(0.001)	−0.95(0.000)
*lba0223*	*cdpA* – Cell separation protein	0.14 (0.072)	1.44 (0.000)
*lba1512*	*prtP*	0.32 (0.002)	0.08 (0.596)
*lba1599*	*fbaA* – Fructose-bisphosphate aldolase	0.10 (0.144)	−0.78(0.000)
*lba0289*	*fusA* – Elongation factor G	−0.21(0.010)	−0.17(0.000)
*lba1611*	*fmtB* – Surface protein	−0.09(0.364)	1.88 (0.000)
*lba0858*	Penicillin-binding protein	0.48 (0.000)	−0.79(0.001)
*lba0698*	Glyceraldehyde-3-p dehydrogenase	0.09 (0.220)	−0.25(0.000)
*lba0957*	*kpyK* – Pyruvate kinase	−0.04(0.598)	−0.38(0.000)
*lba0845*	*tuf* – Elongation factor Tu	0.00 (0.941)	−0.15(0.000)
*lba1918*	*lysA* – Lysin	0.37 (0.000)	−0.07(0.341)
*lba1763*	*pepF* – Oligopeptidase	−0.26(0.000)	−0.71(0.000)
*lba0185*	*gpmA* – 2,3-bisphosphoglycerate-dependent phosphoglycerate mutase	0.09 (0.190)	−0.92(0.000)
*lba0222*	Putative uncharacterized protein	0.39 (0.000)	1.16 (0.000)
*lba0831*	*bipA* – GTP-binding protein-BipA-EF-TU family	−0.31(0.000)	−0.81(0.000)
*lba1270*	*rpsB* – 30S ribosomal protein S2	−0.34(0.000)	−0.04(0.378)
*lba1225*	Putative uncharacterized protein	0.05 (0.581)	−0.68(0.000)
*lba1262*	*proS* – Proline–tRNA ligase	−0.20(0.005)	0.19 (0.001)
*lba1543*	*thrS* – Threonine–tRNA ligase	0.02 (0.754)	−0.88(0.000)

When subjected to salt stress, the mutant exhibited considerable changes to its expression profile. Significantly upregulated genes included members of a previously characterized two-component regulatory system (Pfeiler et al., 2007), as well as secondary surface proteins SlpX (Goh et al., 2009) and aggregation-promoting factor (Apf) (Goh and Klaenhammer, 2010); downregulated genes predominantly coded for ABC transporters and purine metabolism genes (Figure 7A, right). COG analysis reinforced this data as most of the downregulated genes were assigned to roles in nucleotide and carbohydrate transport and metabolism. Upregulated COGs were predominantly unknown or poorly characterized surface proteins (Figure 7B, right). Notably, the presence of salt had no effect on *idgA* expression within the parent strain.

The significantly up and downregulated genes identified in Figure 7A were also found to belong to several operons which are depicted in Supplementary Figure S3. This same subset of differentially expressed genes was analyzed using the DAVID algorithm (Huang et al., 2009b, a) to detect enriched functional categories and KEGG pathways. The results revealed both the induction and repression of several carbohydrate transporter and metabolism genes in both MRS broth and the high salt medium (Supplementary Figure S4).

## Discussion

S-layers are comprised of highly abundant surface molecules which exhibit broad functionalities in both pathogenic and probiotic bacteria. Despite their characterized roles in host immunomodulation (Konstantinov et al., 2008; Suzuki et al., 2019) and intestinal adhesion (Antikainen et al., 2002; Buck et al., 2005), S-layers have only been experimentally detected on the surfaces of a select few species within the *Lactobacillus* genus (Hynonen and Palva, 2013). These results are consistent with our own *in silico* findings which underscored just how relatively uncommon S-layer-forming lactobacilli are. Indeed, less than a quarter of the 170 analyzed strains, representing 170 species, possessed Slp-encoding genes. Those strains which did carry them were either host-adapted (vertebrate or insect) or free-living species. Interesting was the polarity created by the presence of *igdA*. The gene was only detected in S-layer-forming strains, as previously reported (Johnson et al., 2015), but restricted to host-adapted species, indicating a possible host-related function. This relationship was further teased apart when it was revealed that within these host-adapted *igdA*-containing strains, the Ig domain itself was unique to vertebrate-adapted species. It should be noted that the lifestyle data was modified from the original publication (Duar et al., 2017) to re-classify *L. acidophilus* NCFM as vertebrate-adapted, for which there is ample evidence (Sanders and Klaenhammer, 2001; Altermann et al., 2005; Celebioglu et al., 2017). Findings on the vertebrate host-specificity of *igdA* prompted further investigation into the potential probiotic attributes of this gene feature.

The *lba0695* locus, encoding SLAP IgdA, was deleted from the *L. acidophilus* NCFM chromosome. The removal of this gene had considerable impact on host cellular physiology and probiotic functionality. An initial survey of the mutant exoproteome revealed excess surface protein that neither correlated with changes in expression nor relative abundancies. This unusual shedding, in addition to the severe, substrate-independent, reductions in adherence, served as early indications that the *igdA*-deficient strain possessed an irregular cell surface structure.

*Lactobacillus acidophilus* Slps are known to anchor to the cell exterior through interactions with lipoteichoic acid (LTA), a surface-associated adhesion amphiphile (Antikainen et al., 2002; Fina Martin et al., 2019). Increased Slp shedding by Δ*igdA* suggests not only a function in S-layer formation, but also a probable relationship with LTA, though notably, *ltaS* expression was unaffected by the deletion. However, like Δ*igdA*, an LTA-deficient strain of *L. acidophilus* exhibited elongated cells mediated through aberrant cell division and attributed to the upregulation of peptidoglycan turnover genes such as lysin (Selle et al., 2017). Similarly, *L. acidophilus* with an insertionally inactivated cell division protein, *cdpA*, also developed elongated cells, salt sensitivity, and an altered cell wall structure (Altermann et al., 2004). Notably, CdpA is predicted to have an Ig/albumin-binding domain.

Despite the increased protein isolated via LiCl treatment, expression of all three Slp constituents (SlpA, SlpB, and SlpX), and predominate log phase SLAPs, was unaffected by the *igdA* deletion. Further transcriptional analyses revealed the induction of several stress response genes and secondary surface proteins. Although TEM images did not yield conclusive evidence regarding the state of the mutant cell wall, the upregulation of lysine biosynthesis pathway genes may signify an increased compensation for cell wall strength, as lysine is an important component of peptidoglycan cross-linking (Chapot-Chartier and Kulakauskas, 2014; Kim et al., 2015).

In general, S-layers are known to protect cells against hostile environmental agents, and are selectively expressed during unfavorable growth conditions (Khaleghi et al., 2010; Palomino et al., 2016). A previous study which examined salt-stressed *L. acidophilus* noted increases in SlpA and SlpX expression, as well as the release of S-layer proteins into the supernatant (Palomino et al., 2016). Likewise, an *slpA* mutant strain of *L. acidophilus* NCFM was reported to be osmosensitive (Klaenhammer et al., 2005). Within salt-stressed Δ*igdA* cells, genes coding for surface proteins SlpX and Apf were strikingly induced compared to the parent strain. The excessive upregulation of *apf* could explain the kinked cellular morphology observed with phase contrast microscopy and SEM. In *Lactobacillus gasseri* HB2, synthetic overproduction of Apf proteins resulted in a similar phenotype (Jankovic et al., 2003). Though *L. gasseri* does not produce an S-layer, its Apf proteins are considered S-layer-like as they share many similarities to Slps including relative abundance, LiCl extractability, amino acid composition, and predicted physical properties such as a high pI (Ventura et al., 2002). In addition to surface proteins, salt stress also triggered the upregulation of a previously characterized bile-inducible operon encoding a two-component regulatory system (Pfeiler et al., 2007). This system includes two-component response regulator, LBA1431, which possess an OmpR/PhoB-type DNA-binding domain (Pfeiler et al., 2007) and putative SLAP LBA1426 (Klotz et al., 2017). Interestingly, this cluster of genes was not induced in the salt-stressed parent strain. Overall, it appears that the dysregulated surface structure, coupled with high salt conditions, prompted the overexpression of natural osmoprotective defenses by the *igdA* mutant.

## Conclusion

The deletion of *igdA* in *L. acidophilus* produced significant changes to the cell exterior which contributed to its higher salt sensitivity, reduced adhesive capacity, and altered immunogenicity profile. Though the underlying mechanism of IgdA is still uncertain, the aforementioned phenotypes were presumed to be a pleiotropic response resulting from a disordered surface structure. Nevertheless, it is clear that IgdA is necessary for preserving *L. acidophilus* cell surface integrity and possibly S-layer array formation capabilities. Considering the predicted role of Ig domains in host-adaption, as well as the broad impact of IgdA on the cellular physiology of *L. acidophilus*, the identification of similar proteins in other bacteria may help inform next-generation probiotic screening efforts.

## Data Availability Statement

The datasets generated for this study can be found in the National Center for Biotechnology database under BioProject ID PRJNA576881.

## Author Contributions

CK, YG, SO’F, and RB designed the study. CK carried out the work, analyzed the results, and prepared the manuscript under the advisement of RB, YG, SO’F, and BJ.

## Conflict of Interest

The authors declare that the research was conducted in the absence of any commercial or financial relationships that could be construed as a potential conflict of interest.

## Supplementary Material

FIGURE S1 Growth analysis of *igdA* deletion mutant. Growth of the *igdA* deletion mutant (NCK2532, red) was compared to the parent strain (NCK1909, blue) in MRS broth, and MRS broth supplemented with 2.5% NaCl, 0.5% oxgall, or 0.2% porcine bile. Strains were grown in microtiter plates and the OD_600_ was recorded every hour for 30 h. Each plot is a representative sample from at least three biological replicates.

FIGURE S2 Transmission electron microscopy images of *L. acidophilus* strains. Images depict the parent strain (NCK1909) and *igdA* deletion mutant (NCK2532) grown in MRS broth for 6 and 12 h.

FIGURE S3 Transcriptomic data mapped to the *L. acidophilus* NCFM chromosome. Plots depict Log2 fold change in expression of NCK2532 vs. NCK1909 grown in MRS broth [**(A)**, top] and MRS broth +2.5% NaCl [**(A)**, bottom]. Significantly up and downregulated genes (*p*-value < 0.01 and |Log2 fold change| >1) are colored in red and blue, respectively. Genes that are part of operons are indicated by a filled circle. Operons are numbered and correspond to the genes listed in **(B)**.

FIGURE S4 Functional categories and KEGG pathways of differentially expressed genes. The parent and *igdA* mutant strains were grown in MRS broth and MRS broth supplemented with 2.5% NaCl. Significantly up and downregulated genes (*p*-value < 0.01 and |Log2 fold change| >1) were assigned functional categories and/or KEGG pathways via the DAVID algorithm using default parameters.

TABLE S1 List of the 170 L*actobacillus* strains, lifestyles, and isolation sources.

TABLE S2 Differential expression of *igdA* mutant compared to parent strain when cultured in MRS.

TABLE S3 Differential expression of *igdA* mutant compared to parent strain when cultured in MRS +2.5% NaCl.
