# Supplementary figures and images for: Deletion of S-Layer Associated Ig-Like Domain Protein Disrupts the Lactobacillus acidophilus Cell Surface
Source: Front Microbiol. 2020 Mar 17;11:345. doi: 10.3389/fmicb.2020.00345 (PMC7090030; doi:10.3389/fmicb.2020.00345)

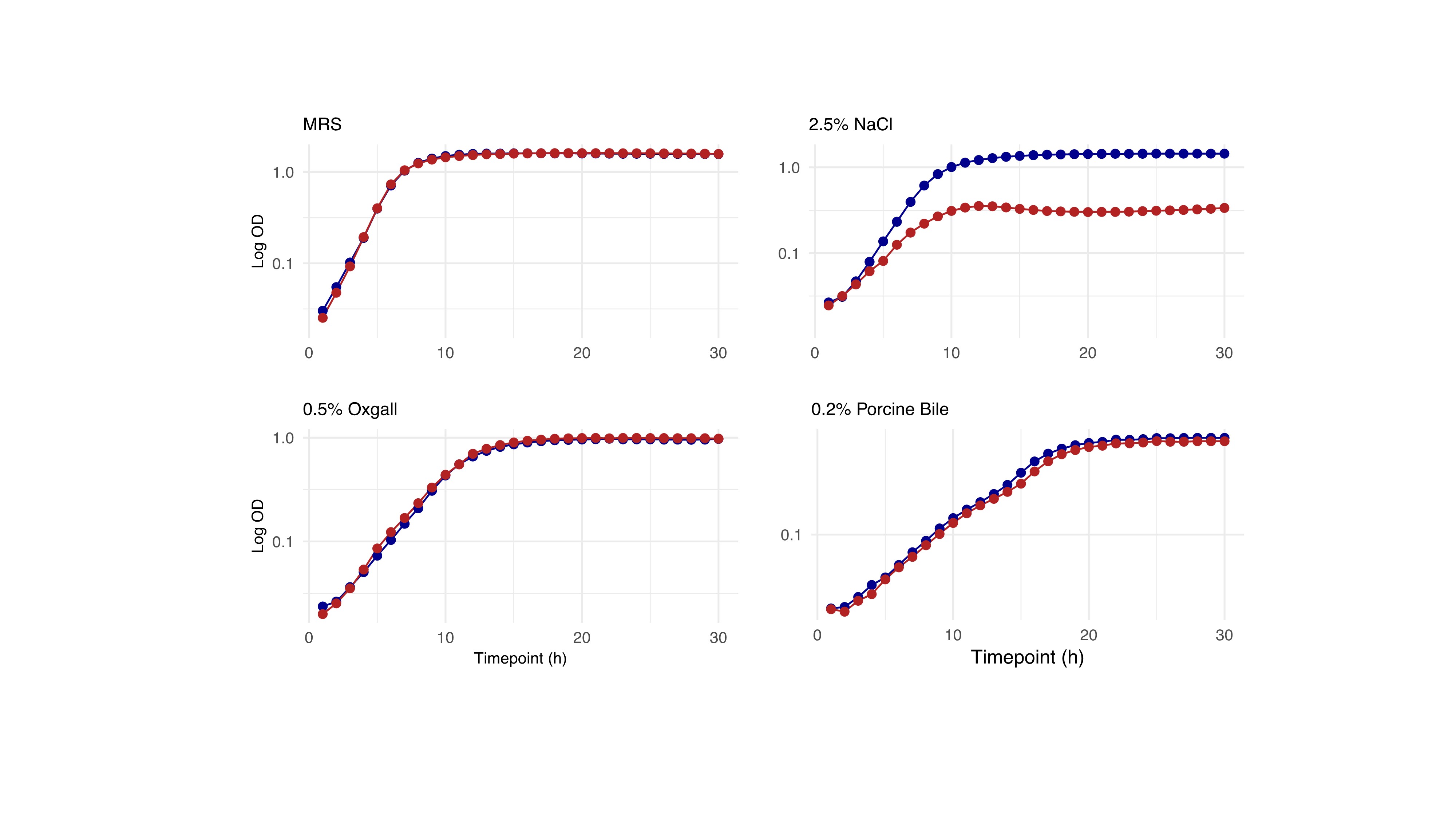

Supplement: FIGURE S1 — Growth analysis of igdA deletion mutant. Growth of the igdA deletion mutant (NCK2532, red) was compared to the parent strain (NCK1909, blue) in MRS broth, and MRS broth supplemented with 2.5% NaCl, 0.5% oxgall, or 0.2% porcine bile. Strains were grown in microtiter plates and the OD600 was recorded every hour for 30 h. Each plot is a representative sample from at least three biological replicates. [file Image_1.JPEG]

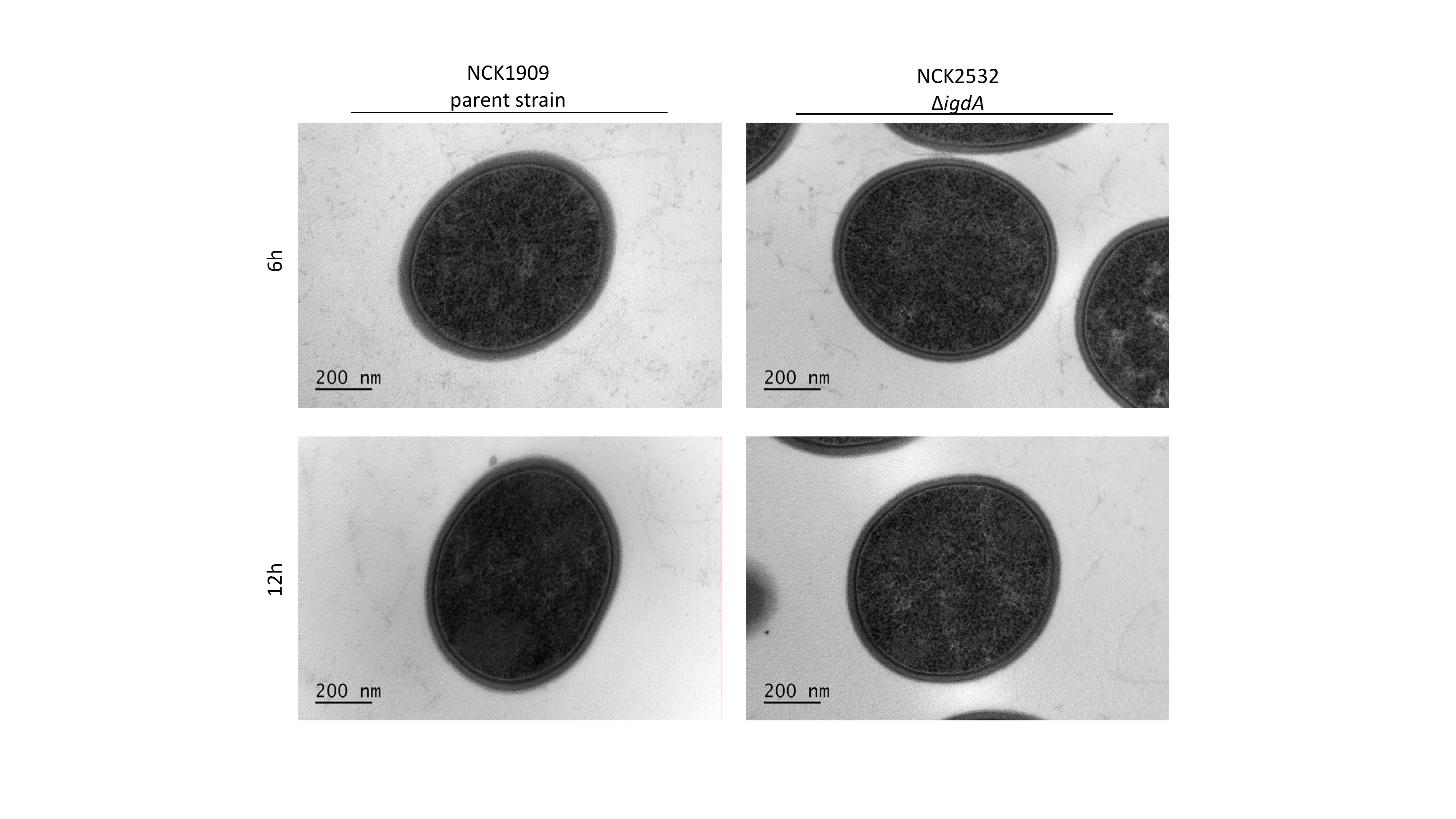

Supplement: FIGURE S2 — Transmission electron microscopy images of L. acidophilus strains. Images depict the parent strain (NCK1909) and igdA deletion mutant (NCK2532) grown in MRS broth for 6 and 12 h. [file Image_2.JPEG]

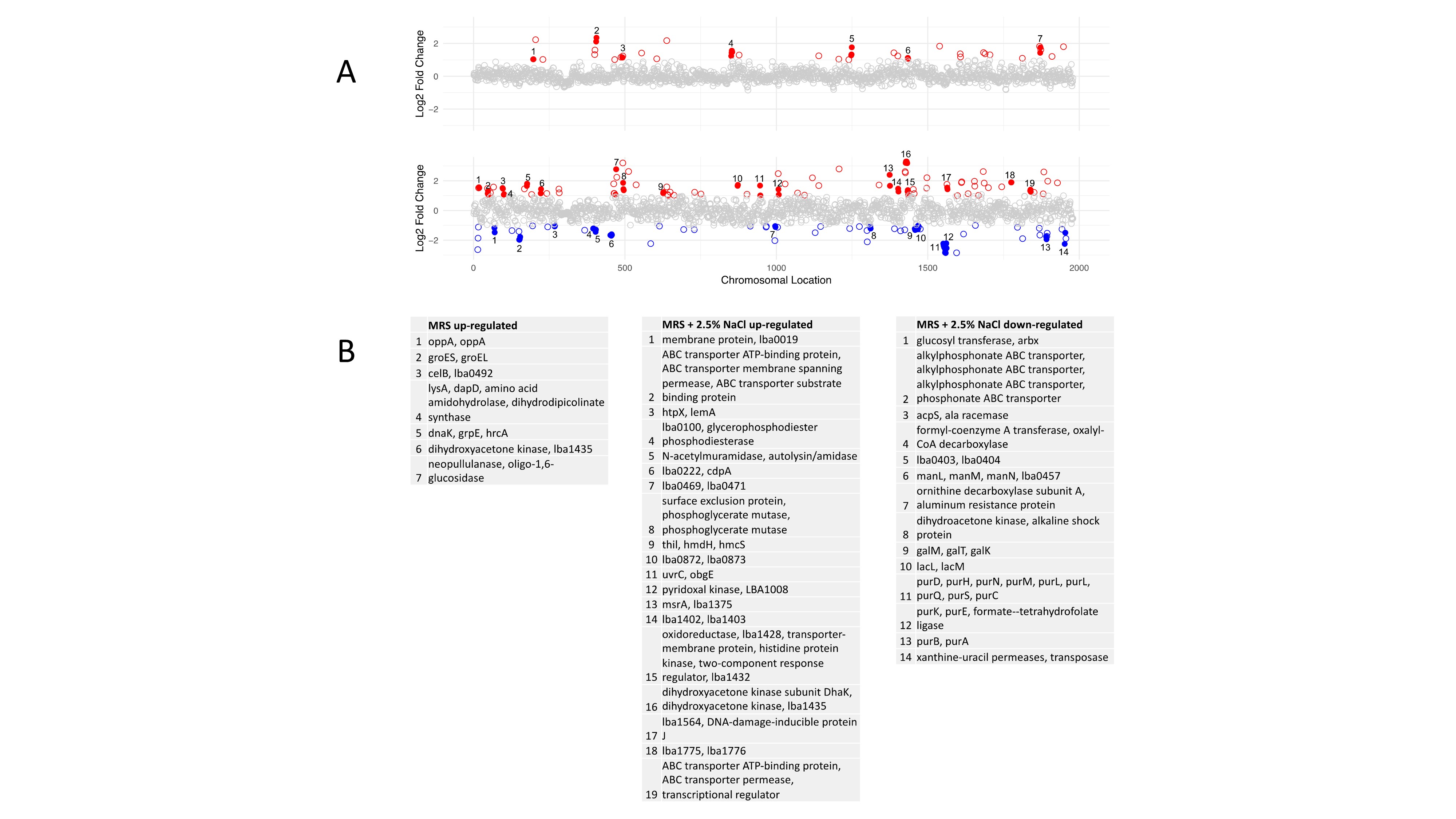

Supplement: FIGURE S3 — Transcriptomic data mapped to the L. acidophilus NCFM chromosome. Plots depict Log2 fold change in expression of NCK2532 vs. NCK1909 grown in MRS broth [(A), top] and MRS broth +2.5% NaCl [(A), bottom]. Significantly up and downregulated genes (p-value < 0.01 and |Log2 fold change| >1) are colored in red and blue, respectively. Genes that are part of operons are indicated by a filled circle. Operons are numbered and correspond to the genes listed in (B). [file Image_3.JPEG]

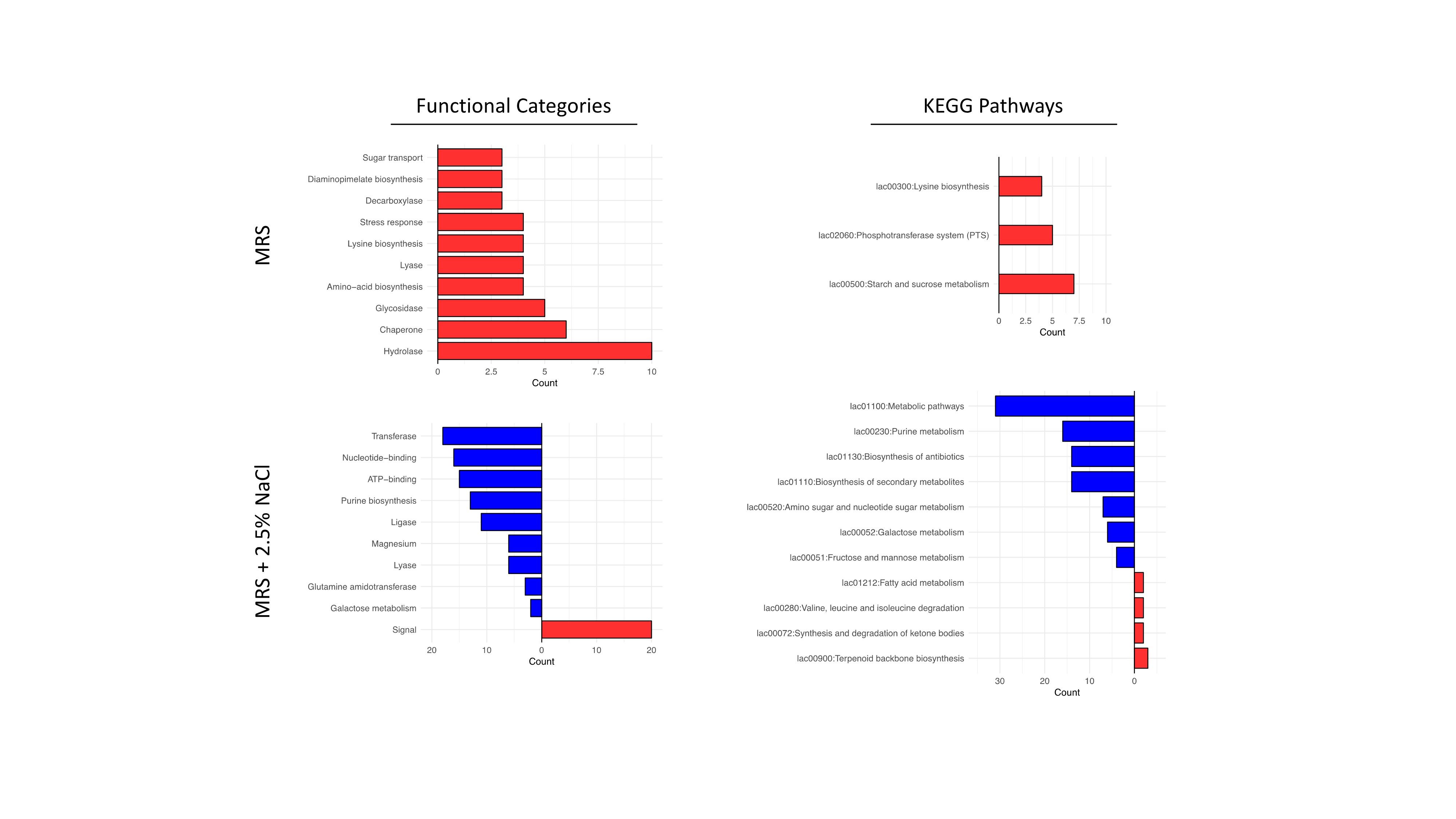

Supplement: FIGURE S4 — Functional categories and KEGG pathways of differentially expressed genes. The parent and igdA mutant strains were grown in MRS broth and MRS broth supplemented with 2.5% NaCl. Significantly up and downregulated genes (p-value < 0.01 and |Log2 fold change| >1) were assigned functional categories and/or KEGG pathways via the DAVID algorithm using default parameters. [file Image_4.JPEG]
