# Supplementary material for: Deletion of S-Layer Associated Ig-Like Domain Protein Disrupts the Lactobacillus acidophilus Cell Surface
Source: Front Microbiol. 2020 Mar 17;11:345. doi: 10.3389/fmicb.2020.00345 (PMC7090030; doi:10.3389/fmicb.2020.00345)
Supplement: TABLE S1 — List of the 170 Lactobacillus strains, lifestyles, and isolation sources. [file Table_1.DOCX]

**Supplementary Table 1 |** List of the 170 L*actobacillus* strains, lifestyles, and isolation sources.

| ***Lactobacillus* strain** | **Metadata lifestyle** | **Isolation source** |
| --- | --- | --- |
| *L. acetotolerans* NBRC 13120 |  | sake |
| *L. acidifarinae* DSM 19394 |  | artisanal wheat sourdough |
| *L. acidipiscis* KCTC 13900 | Vertebrate-adapted | cheese |
| *L. acidophilus* NCFM | Vertebrate-adapted | human gastrointestinal tract |
| *L. agilis* DSM 20509 | Vertebrate-adapted | municipal sewage |
| *L. algidus* DSM 15638 |  | vacuum-packaged beef |
| *L. alimentarius* DSM 20249 |  | marinated fish product |
| *L. amylolyticus* DSM 11664 | Vertebrate-adapted | acidified beer wort |
| *L. amylophilus* DSM 20533 |  | swine waste-corn fermentation |
| *L. amylotrophicus* DSM 20534 |  | swine waste-corn fermentation |
| *L. amylovorus* 30SC | Vertebrate-adapted | porcine ileum |
| *L. animalis* DSM 20602 | Vertebrate-adapted | dental plaque of baboon |
| *L. antri* DSM 16041 | Vertebrate-adapted | gastric biopsies, human stomach mucosa |
| *L. apinorum* Fhon13 | Insect-adapted | honey stomach of honey bee (Apis mellifera mellifera) |
| *L. apis* Hma11 | Insect-adapted | honey stomach of honey bee |
| *L. apodemi* DSM 16634 | Vertebrate-adapted | feces, wild Japanese wood mouse |
| *L. aquaticus* DSM 21051 | Free-living | surface of a eutrophic freshwater pond |
| *L. aviarius* subsp. aviarius DSM 20655 | Vertebrate-adapted | feces of chicken |
| *L. backii* TMW 1.1988 |  | light wheat beer in German brewery |
| *L. bifermentans* DSM 20003 |  | blown cheese |
| *L. bombicola* R-53102 | Insect-adapted | bumblebee gut (Bombus lapidarius) |
| *L. brantae* DSM 23927 |  | feces of Canada goose (Branta canadensis) |
| *L. brevis* ATCC 367 | Free-living | silage |
| *L. buchneri* CD034 | Free-living | grass silage |
| *L. cacaonum* DSM 21116 |  | cocoa bean heap fermentation |
| *L. camelliae* DSM 22697 |  | fermented tea leaves (miang) |
| *L. capillatus* DSM 19910 |  | fermented brine used for stinky tofu production |
| *L. casei* subsp. casei ATCC 393 | Nomadic | cheese |
| *L. ceti* DSM 22408 | Vertebrate-adapted | lungs of a beaked whale (Ziphius cavirostris) |
| *L. coleohominis* 101-4-CHN | Vertebrate-adapted | human vagina |
| *L. collinoides* DSM 20515 | Free-living | fermenting apple juice |
| *L. composti* DSM 18527 | Free-living | composting material of distilled shochu residue |
| *L. concavus* DSM 17758 |  | distilled pirit-fermenting cellar |
| *L. coryniformis* subsp. coryniformis DSM 20001 |  | silage |
| *L. crispatus* ST1 | Vertebrate-adapted | chicken crop |
| *L. crustorum* MN047 |  | koumiss |
| *L. curieae* CCTCC M 2011381 |  | fermented brine used for stinky tofu production |
| *L. curvatus* FBA2 | Free-living | radish/carrot pickles |

**Supplementary Table 1 |** (continued).

| ***Lactobacillus* strain** | **Metadata lifestyle** | **Isolation source** |
| --- | --- | --- |
| *L. delbrueckii* subsp. bulgaricus ATCC 11842 |  | dairy products; Bulgarian yogurt |
| *L. dextrinicus* DSM 20335 | Free-living | silage |
| *L. diolivorans* DSM 14421 | Free-living | maize silage |
| *L. equi* DSM 15833 | Vertebrate-adapted | feces of horses |
| *L. equicursoris* DSM 19284 | Vertebrate-adapted | healthy thoroughbred racehorse |
| *L. equigenerosi* DSM 18793 | Vertebrate-adapted | thoroughbred horses |
| *L. fabifermentans* DSM 21115 | Nomadic | cocoa bean heap fermentation |
| *L. farciminis* DSM 20184 |  | sausage |
| *L. farraginis* DSM 18382 | Free-living | composting material of distilled shochu residue |
| *L. fermentum* IFO 3956 |  | fermented plant material |
| *L. floricola* DSM 23037 | Free-living | flower of Caltha palustris |
| *L. florum* DSM 22689 | Insect-adapted | peony (Paeonia suffruticosa) |
| *L. fructivorans* DSM 20203 | Insect-adapted | N/A |
| *L. frumenti* DSM 13145 | Vertebrate-adapted | rye-bran sourdough |
| *L. fuchuensis* DSM 14340 | Free-living | vacuum-packaged beef |
| *L. futsaii* JCM 17355 |  | fu-tsai, a traditional fermented mustard product |
| *L. gallinarum* HFD4 | Vertebrate-adapted | chicken crop |
| *L. gasseri* ATCC 33323 | Vertebrate-adapted | human |
| *L. gastricus* DSM 16045 | Vertebrate-adapted | gastric biopsies, human stomach mucosa |
| *L. ghanensis* DSM 18630 |  | cocoa fermentation |
| *L. gigeriorum* DSM 23908 | Vertebrate-adapted | chicken crop |
| *L. ginsenosidimutans* EMML 3041 |  | kimchi |
| *L. gorillae* KZ01 | Vertebrate-adapted | western lowland gorilla (Gorilla gorilla gorilla) |
| *L. graminis* DSM 20719 | Free-living | grass silage |
| *L. hammesii* DSM 16381 | Free-living | wheat sourdough |
| *L. hamsteri* DSM 5661 | Vertebrate-adapted | feces of hamster |
| *L. harbinensis* DSM 16991 | Free-living | chinese traditional fermented vegetable Suan cai |
| *L. hayakitensis* DSM 18933 | Vertebrate-adapted | feces of thoroughbred horse |
| *L. heilongjiangensis* DSM 28069 |  | Chinese traditional pickle |
| *L. helsingborgensis* Bma5 | Insect-adapted | honey stomach of honey bee (Apis mellifera mellifera) |
| *L. helveticus* CNRZ32 | Vertebrate-adapted | artisanal starter, Comte´ cheese |
| *L. herbarum* TCF032-E4 | Nomadic | Chinese fermented radish |
| *L. hilgardii* ATCC 8290 | Free-living | wine |
| *L. hokkaidonensis* JCM 18461 | Free-living | subarctic timothy grass (Phleum pratense L.) |
| *L. hominis* DSM 23910 | Vertebrate-adapted | human intestine |
| *L. homohiochii* DSM 20571 | Insect-adapted | spoiled sake |
| *L. hordei* DSM 19519 | Free-living | malted barley |
| *L. iners* DSM 13335 | Vertebrate-adapted | human urine |
| *L. ingluviei* str. Autruche 4 | Vertebrate-adapted | ostrich |

**Supplementary Table 1 |** (continued).

| ***Lactobacillus* strain** | **Metadata lifestyle** | **Isolation source** |
| --- | --- | --- |
| *L. intestinalis* DSM 6629 | Vertebrate-adapted | intestine of rat |
| *L. jensenii* SNUV360 | Vertebrate-adapted | human vagina |
| *L. johnsonii* NCC 533 | Vertebrate-adapted | human isolate |
| *L. kalixensis* DSM 16043 | Vertebrate-adapted | gastric biopsies, human stomach mucosa |
| *L. kefiranofaciens* ZW3 |  | Tibet kefir |
| *L. kefiri* DSM 20587 | Free-living | kefir grains |
| *L. kimbladii* Hma2 | Insect-adapted | honey stomach of honey bee (Apis mellifera) |
| *L. kimchicus* JCM 15530 |  | kimchi |
| *L. kimchiensis* DSM 24716 |  | kimchi |
| *L. kisonensis* DSM 19906 | Free-living | non-salted pickle solution used in production of sunki |
| *L. kitasatonis* DSM 16761 | Vertebrate-adapted | chicken intestine |
| *L. koreensis* 26-25 | Free-living | kimchi |
| *L. kullabergensis* Biut2 | Insect-adapted | honey stomach of honey bee (Apis mellifera mellifera) |
| *L. kunkeei* MP2 | Insect-adapted | guts of Chilean honey bees (Apis mellifera) |
| *L. lindneri* TMW 1.481 |  | brewery environment |
| *L. malefermentans* DSM 5705 |  | beer |
| *L. mali* ATCC 27304 | Free-living | wine must, Japan |
| *L. manihotivorans* DSM 13343 |  | cassava sour starch fermentation |
| *L. mellifer* Bin4 | Insect-adapted | Apis mellifera Buckfast (honeybee) |
| *L. mellis* Hon2 | Insect-adapted | Apis mellifera Buckfast (fresh honey) |
| *L. melliventris* Hma8 | Insect-adapted | Apis mellifera Buckfast (honey stomach) |
| *L. mindensis* DSM 14500 |  | sourdough |
| *L. mucosae* LM1 | Vertebrate-adapted | stool samples of a healthy piglet |
| *L. murinus* DSM 20452 | Vertebrate-adapted | intestine of rat |
| *L. nagelii* DSM 13675 |  | partially fermented wine |
| *L. namurensis* DSM 19117 | Free-living | sourdough, manufactured with wheat, rye and spelt flour |
| *L. nantensis* DSM 16982 |  | wheat sourdough |
| *L. nasuensis* JCM 17158 |  | sudangrass silage sample |
| *L. nodensis* DSM 19682 |  | Japanese pickles |
| *L. odoratitofui* DSM 19909 |  | fermented brine used for stinky tofu production |
| *L. oeni* DSM 19972 |  | Bobal wine |
| *L. oligofermentans* DSM 15707 | Free-living | broiler leg |
| *L. oris* DSM 4864 | Vertebrate-adapted | human saliva |
| *L. oryzae* JCM 18671 |  | fermented rice grain (Oryza sativa L. subsp. japonica) |
| *L. otakiensis* DSM 19908 | Free-living | non-salted pickle solution used in production of sunki |
| *L. ozensis* DSM 23829 | Insect-adapted | Inula ciliaris var. glandulosa, a chrysanthemum |
| *L. panis* DSM 6035 | Vertebrate-adapted | sourdough |
| *L. pantheris* DSM 15945 |  | jaguar feces |

**Supplementary Table 1 |** (continued).

| ***Lactobacillus* strain** | **Metadata lifestyle** | **Isolation source** |
| --- | --- | --- |
| *L. parabrevis* ATCC 53295 | Free-living | cheese |
| *L. parabuchneri* FAM21731 | Free-living | Swiss Emmental cheese |
| *L. paracasei* ATCC 334 | Nomadic | dairy products; emmental cheese |
| *L. paracollinoides* strain TMW 1.1994 | Free-living | brewery environment |
| *L. parafarraginis* DSM 18390 | Free-living | composting material of distilled shochun residue |
| *L. parakefiri* DSM 10551 | Free-living | kefir grain |
| *L. paralimentarius* DSM 13961 |  | kimchi |
| *L. paraplantarum* DSM 10667 | Nomadic | beer contaminant |
| *L. pasteurii* DSM 23907 | Vertebrate-adapted | N/A |
| *L. paucivorans* DSM 22467 | Free-living | yeast storage tank containing lager beer |
| *L. pentosus* DSM 20314 | Nomadic | N/A |
| *L. perolens* DSM 12744 | Free-living | orange lemonade |
| *L. plantarum* WCFS1 | Nomadic | human saliva |
| *L. pobuzihii* E100301 |  | pobuzihi (fermented cummingcordia) |
| *L. pontis* DSM 8475 | Vertebrate-adapted | rye sourdough |
| *L. psittaci* DSM 15354 | Vertebrate-adapted | lung of parrot |
| *L. rapi* DSM 19907 | Free-living | non-salted pickle solution used in production of sunki |
| *L. rennini* DSM 20253 |  | rennin |
| *L. reuteri* DSM 20016 | Vertebrate-adapted | intestine of adult |
| *L. rhamnosus* GG | Nomadic | intestinal tract of a healthy human |
| *L. rogosae* ATCC 27753 |  | human feces |
| *L. rossiae* DSM 15814 |  | wheat sourdough |
| *L. ruminis* ATCC 27782 | Vertebrate-adapted | rumen |
| *L. saerimneri* DSM 16049 | Vertebrate-adapted | pig feces |
| *L. sakei* subsp. sakei 23K | Free-living | French sausage |
| *L. salivarius* UCC118 | Vertebrate-adapted | human ileal-cecal region |
| *L. sanfranciscensis* TMW 1.1304 | Insect-adapted | sourdough, commercial mother sponge |
| *L. saniviri* JCM 17471 |  | feces of a Japanese healthy adult male |
| *L. satsumensis* DSM 16230 |  | shochu mash |
| *L. secaliphilus* DSM 17896 |  | sour dough |
| *L. selangorensis* DSM 13344 |  | chili bo |
| *L. senioris* DSM 24302 | Vertebrate-adapted | feces of a healthy 100-year-old Japanese female |
| *L. senmaizukei* DSM 21775 | Free-living | pickles |
| *L. sharpeae* DSM 20505 |  | municipal sewage |
| *L. shenzhenensis* LY-73 | Free-living | fermented beverage |
| *L. silagei* JCM 19001 |  | orchardgrass silage, |
| *L. siliginis* DSM 22696 |  | wheat sourdough |
| *L. similis* DSM 23365 | Free-living | fermented cane molasses at alcohol plants |
| *L. spicheri* DSM 15429 | Free-living | rice sourdough |
| *L. sucicola* DSM 21376 | Free-living | sap of Quercus sp. |

**Supplementary Table 1 |** (continued).

| ***Lactobacillus* strain** | **Metadata lifestyle** | **Isolation source** |
| --- | --- | --- |
| *L. suebicus* DSM 5007 | Free-living | apple mash |
| *L. sunkii* DSM 19904 | Free-living | non-salted pickle solution used in production of sunki |
| *L. taiwanensis* DSM 21401 | Vertebrate-adapted | silage cattle feed |
| *L. thailandensis* DSM 22698 |  | fermented tea leaves (miang) |
| *L. tucceti* DSM 20183 |  | sausage |
| *L. ultunensis* DSM 16047 | Vertebrate-adapted | gastric biopsies, human stomach mucosa |
| *L. uvarum* DSM 19971 | Free-living | must of Bobal grape variety |
| *L. vaccinostercus* DSM 20634 | Free-living | cow dung |
| *L. vaginalis* DSM 5837 | Vertebrate-adapted | vaginal swab from patient with trichomoniasis |
| *L. versmoldensis* DSM 14857 |  | poultry salami |
| *L. vini* DSM 20605 |  | grape must, fermenting at high temperature |
| *L. wasatchensis* WDC04 | Free-living | aged Cheddar cheese |
| *L. xiangfangensis* LMG 26013 |  | pickle |
| *L. zeae* DSM 20178 |  | corn steep liquor |
| *L. zymae* DSM 19395 | Free-living | artisanal wheat sourdough |
